# Impaired Efficiency and Resilience of Structural Network in Spinocerebellar Ataxia Type 3

**DOI:** 10.3389/fnins.2018.00935

**Published:** 2018-12-17

**Authors:** Yu-Te Wu, Shang-Ran Huang, Chi-Wen Jao, Bing-Wen Soong, Jiing-Feng Lirng, Hsiu-Mei Wu, Po-Shan Wang

**Affiliations:** ^1^Department of Biomedical Imaging and Radiological Sciences, National Yang-Ming University, Taipei, Taiwan; ^2^Institute of Biophotonics and Brain Research Center, National Yang-Ming University, Taipei, Taiwan; ^3^Department of Neurology, Shuang Ho Hospital and Taipei Neuroscience Institute, Taipei Medical University, Taipei, Taiwan; ^4^Department of Neurology, Taipei Veterans General Hospital and Brain Research Center, National Yang-Ming University, Taipei, Taiwan; ^5^Department of Medicine, School of Medicine, National Yang-Ming University, Taipei, Taiwan; ^6^Department of Radiology, Taipei Veterans General Hospital, Taipei, Taiwan; ^7^Department of Neurology, Taipei Municipal Gan-Dau Hospital, Taipei, Taiwan

**Keywords:** brain connectivity, fractal dimension, graph theoretical analysis, spinocerebellar ataxia type 3, structural network

## Abstract

**Background:** Recent studies have shown that the patients with spinocerebellar ataxia type 3 (SCA3) may not only have disease involvement in the cerebellum and brainstem but also in the cerebral regions. However, the relations between the widespread degenerated brain regions remains incompletely explored.

**Methods:** In the present study, we investigate the topological properties of the brain networks of SCA3 patients (*n* = 40) constructed based on the correlation of three-dimensional fractal dimension values. Random and targeted attacks were applied to measure the network resilience of normal and SCA3 groups.

**Results:** The SCA3 networks had significantly smaller clustering coefficients (*P* < 0.05) and global efficiency (*P* < 0.05) but larger characteristic path length (*P* < 0.05) than the normal controls networks, implying loss of small-world features. Furthermore, the SCA3 patients were associated with reduced nodal betweenness (*P* < 0.001) in the left supplementary motor area, bilateral paracentral lobules, and right thalamus, indicating that the motor control circuit might be compromised.

**Conclusions:** The SCA3 networks were more vulnerable to targeted attacks than the normal controls networks because of the effects of pathological topological organization. The SCA3 revealed a more sparsity and disrupted structural network with decreased values in the largest component size, mean degree, mean density, clustering coefficient, and global efficiency and increased value in characteristic path length. The cortico-cerebral circuits in SCA3 were disrupted and segregated into occipital-parietal (visual-spatial cognition) and frontal-pre-frontal (motor control) clusters. The cerebellum of SCA3 were segregated from cerebellum-temporal-frontal circuits and clustered into a frontal-temporal cluster (cognitive control). Therefore, the disrupted structural network presented in this study might reflect the clinical characteristics of SCA3.

## Introduction

SCA3 is a hereditary neurodegenerative disorder caused by an aberrant gene mutation (CAG expansion) in chromosome 14q32 (do Carmo Costa and Paulson, 2012). Progressive ataxia, external ophthalmoplegia, dysarthria, dysphagia, pyramidal signs, dystonia, rigidity, and peripheral neuropathy are characteristic symptoms of SCA3 (do Carmo Costa and Paulson, 2012). Many neuropathology (Durr et al., 1996; Koeppen, 2005; Rüb et al., 2008; Scherzed et al., 2012; Takiyama et al., 2012) and neuroimaging studies (Klockgether et al., 1998; Wang et al., 2007, 2012a; Schulz et al., 2010; Eichler et al., 2011; D'Abreu et al., 2012; Guimaraes et al., 2013; Reetz et al., 2013; Adanyeguh et al., 2015; Stefanescu et al., 2015) have reported structural and functional degeneration in the brainstem and cerebellum (cortex, vermis, peduncles, and deep nuclei), which can explain the occurrence of most ataxia symptoms in SCA3 patients. Moreover, many related studies have demonstrated that SCA3 patients may exhibit varieties of non-cerebellum-related symptoms (Pedroso et al., 2013). These results have revealed that cerebral disease also influences the clinical characteristics of SCA3. Therefore, a better understanding of the effects of cerebral degeneration on the brains of SCA3 patients is crucial. Thus far, most studies have only reported degeneration in separate cerebral regions, and global investigation studies, such as brain network analyses, are scant.

In contemporary neuroscience, the materialization of many brain functions relies on dynamical connections between various brain regions (Catani et al., 2012). The functional and structural brain connections can be considered a brain network-“the connectome” (Sporns et al., 2005). Graph theoretical analysis provides a powerful framework for describing the topology of structural or functional brain networks (Rubinov and Sporns, 2010). Several network properties, including the characteristic path length, clustering coefficient, global efficiency, and betweenness centrality, can indicate the integration, segregation, and centrality of a brain network (Aerts et al., 2016). Changes in the properties of structural or functional brain networks have been observed in several neurological, developmental, and psychiatric disorders through graph theoretical analysis (Braun et al., 2009; Damien et al., 2009; Griffa et al., 2013; van Straaten and Stam, 2013; Cao et al., 2015). The maturing of human brain revealed a trend toward segregation (decrease in correlation strength) between regions close in anatomical space and integration (increase in correlation strength) between selected regions distant in space. The organization of multiple functional networks shifts from a local anatomical emphasis in children to a more distributed architecture in young adults (Damien et al., 2009). Moreover, network resilience, a property employed to estimate the ability of a network to withstand damage, can be used to evaluate network organization in addition to the aforementioned general network properties (Aerts et al., 2016). Random attacks and targeted attacks are two common approaches for evaluating network resilience (Albert et al., 2000). Some studies have demonstrated alterations in network resilience in some neurological and psychiatric disorders (He et al., 2008; Arzouan et al., 2014; Jiang et al., 2016; Mak et al., 2016).

In our previous study, we reported that SCA3 patients exhibited disruption of the structural correlation between the parietal-occipital lobes and cerebellar regions (Huang et al., 2017). In that study, the structural correlation of paired brain regions was measured using three-dimensional fractal dimension (3D-FD) values (Huang et al., 2017). The results indicated that cerebral neurodegeneration may disorganise the structural networks of SCA3 patients (Alexander-Bloch et al., 2013; Evans, 2013). Therefore, in the present study, we hypothesized that SCA3 patients would exhibit alterations in the brain structural networks, and we investigated the alterations using graph theoretical analysis. We expected that SCA3 patients would present abnormalities in network parameters, such as characteristic path length, clustering coefficient, and global efficiency, and have altered betweenness centrality of some essential nodes. In addition, the networks of SCA3 patients are anticipated to be more vulnerable to random attacks and targeted attacks.

## Materials and Methods

### Patients and Controls

The study procedures were in accordance with the Declaration of Helsinki and approved by the Institutional Review Board of Taipei Veterans General Hospital. Forty-seven normal subjects (23 males and 24 females) and forty-five SCA3 patients (23 males and 22 females) were recruited in the beginning of this study. Participants of age over 70 or below 30 years old in each group were excluded so that the numbers of male and female in each group and between two groups were the same to minimize the age and gender effects. Additionally, the T1-weighted images of each participant were examined by an experienced neuroradiologist to verify the quality of image. Subjects with blur boundary of atrophied cerebellum or cerebral T1 images were also excluded. Forty SCA3 patients and 40 age- and sex-matched healthy controls participated in this study after exclusion. All participants provided written informed consent that was approved by the Ethics Committee of Taipei Veterans General Hospital. All SCA3 patients were evaluated using the Scale for the Assessment and Rating of Ataxia (Schmitz-Hübsch et al., 2006). A self-reported age at onset was obtained from each patient. The age at onset was defined as the age at which a patient showed the first sign of any ataxic symptom (Jardim et al., 2001). None of the healthy controls exhibited any neurological symptoms before or during the study period. The demographic data of the participants are listed in Table 1. There was no significant difference of age or gender between control and SCA3 groups. The SARA scores for the SCA3 patients revealed that they were in status of walker gait. The mean CAG repeat length for the SCA3 group was 73.2 ± 4.0.

**Table 1:** Demographic data of normal and SCA3 groups.

**Variables**	**Control (*n* = 40)**	**SCA3 (*n* = 40)**
Age (y)	51.20 ± 17.58	45.9 ± 11.9
M/F	20/20	20/20
Age at onset (y)	ND	38.1 ± 10.9
Disease duration (y)	ND	7.7 ± 5.0
SARA score	ND	14.2 ± 8.6
CAG repeat length	ND	73.2 ±4.0

*SARA, Scale for the Assessment and Rating of Ataxia*.

### Image Acquisition

A 1.5-T MRI system was used to obtain the brain images of each participant. The MRI pulse sequence included an axial, T1-weighted, three-dimensional, fast-spoiled, gradient-recalled acquisition of steady state images [repetition time [TR] = 8.58 ms, echo time [TE] = 3.62 ms, inversion time = 400 ms, slice thickness = 1.5 mm, matrix size = 256 × 256, and in-plane resolution of 1 mm × 1 mm], and an axial, T2-weighted, fast spin-echo sequence (TR = 4,000 ms, TE = 256.5 ms, slice thickness = 5 mm).

### Image Pre-processing and 3D-FD Value Computation

Fractal dimension was originally proposed to quantify the complexity of objects with self-similarity (Mandelbrot and Pignoni, 1983). Since Kiselev et al. (2003) reported that the cerebral cortex is self-similar within a certain range of spatial resolutions many studies have used fractal dimension to investigate morphological changes in the cerebral cortex caused by neurological diseases (Ha et al., 2005; Thompson et al., 2005; Sandu et al., 2008; King et al., 2010). Our previous study demonstrated that 3D-FD values exhibited fewer age and gender effects than cortical volume when used for evaluating cortical atrophy in patients with multiple system atrophy-cerebellar type (Wu et al., 2010). In this study, a box-counting algorithm was employed to compute the 3D-FD values of the participants' cerebral and cerebellar cortices on the basis of their T1-weighted images (Zhang et al., 2006).

**Figure 1:**
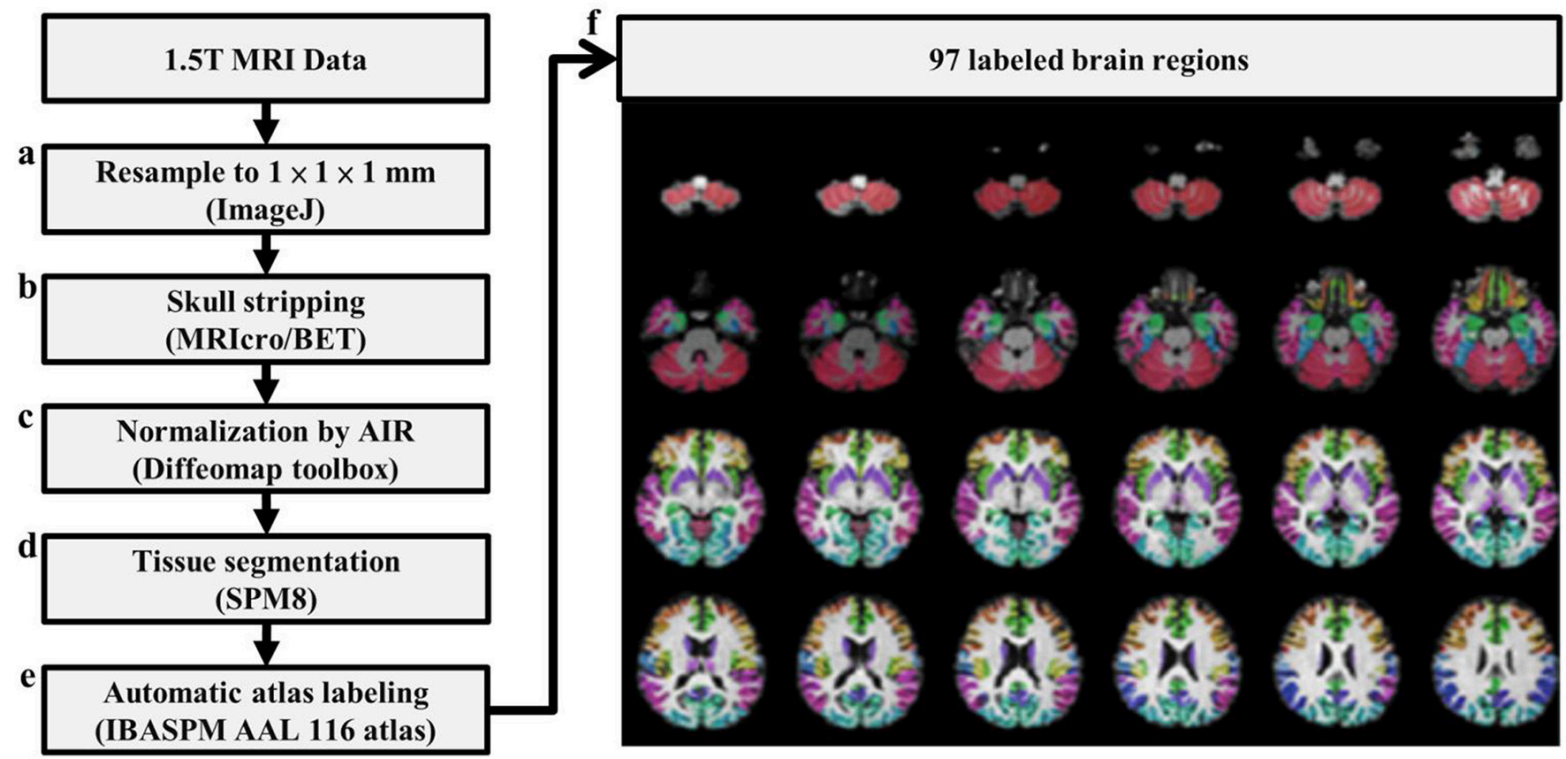
Flowchart of image pre-processing procedure.

Pre-processing was performed before the calculation of 3D-FD values. Figure 1 depicts the flowchart of pre-processing procedures. The spatial resolution of the acquired T1-weighted images was first resampled to 1 × 1 × 1 mm by using ImageJ (Rasband and Image, 1997) (Figure 1A). Before the brain tissue was segmented, skull stripping was performed using the brain extraction tool in MRIcro (Rorden C, University of Nottingham, UK^1^) (Figure 1B). The skull-stripped images were further smoothed by a 2-D median filter with a 3 × 3 kernel on each slice to improve the signal-to-noise ratio (SNR) while preserving the sharpness of the tissue boundary. The skull-stripped and filtered images were then coregistered to the JHU_MNI_SS_T_ss T1 template by a 12-parameter affine transformation in the DiffeoMap toolbox (Li X, Jiang H, and Mori S, Johns Hopkins University, www.mristudio.org) (Figure 1C). Brain segmentation was performed using the SPM8 toolbox ^2^ (Figure 1D). Brain parcellation was performed according to the IBASPM^3^ toolbox in MATLAB R2013b software (Mathworks, Natick, MA, USA). The brain cortex was subsequently parcellated into 116 regions (cerebrum: 90 regions, cerebellum: 26 regions) and labeled using the AAL (Automated Anatomical Labeling) atlas based on IBASPM (Figure 1E). The 26 regions of the cerebellum were then merged into the seven regions, which were the left anterior lobe, right anterior lobe, left posterior upper lobe, right posterior upper lobe, left posterior lower lobe, right posterior lower lobe, and vermis, according to their anatomical structures. Hence, 97 labeled brain regions were obtained for each participant (Figure 1F).

### Graph Theoretical Analysis

**Figure 2:**
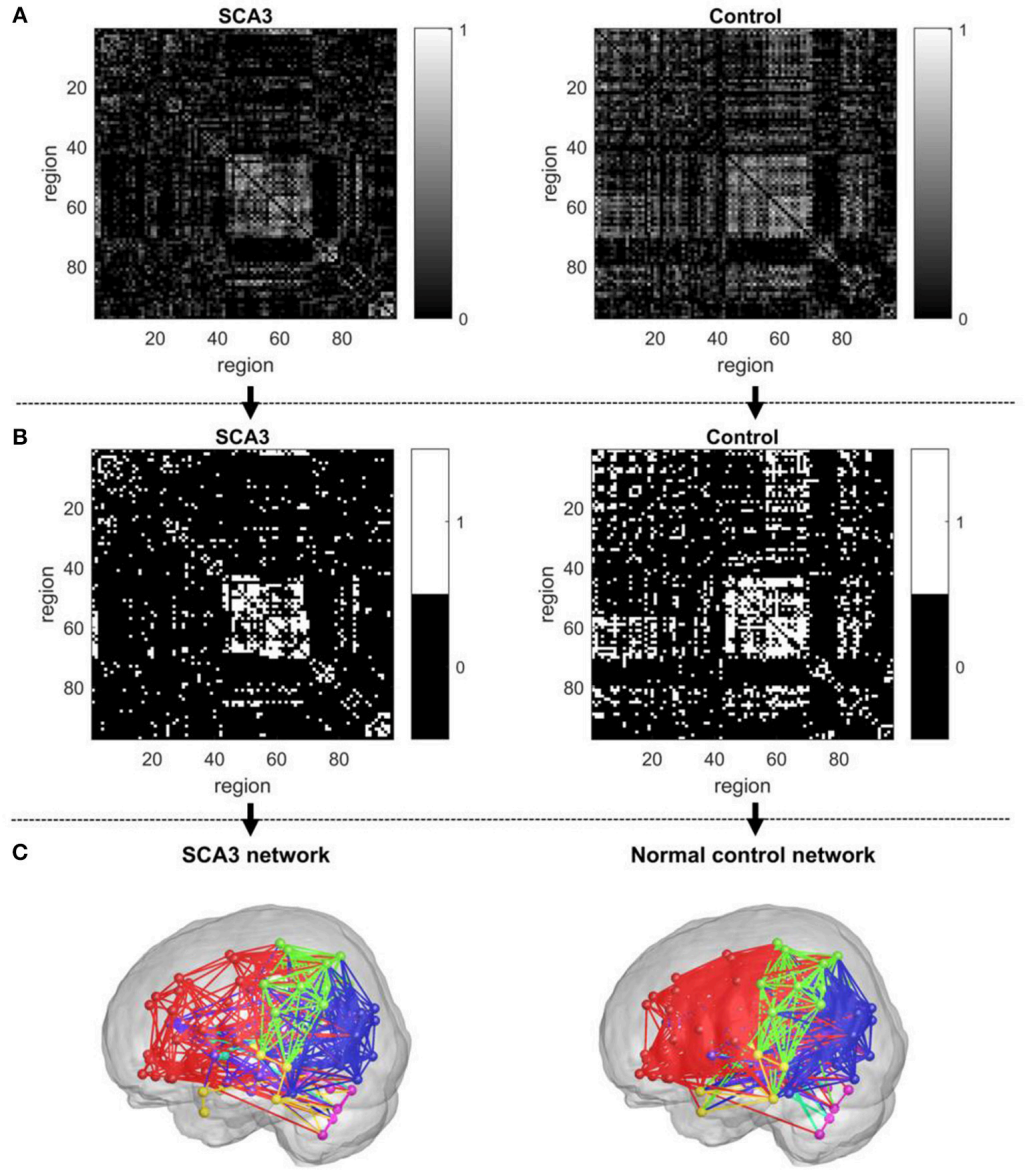
Construction of an unweighted, undirected structural network. **(A)** Correlation matrix of the SCA3 group (left) and control group (right). **(B)** Connection matrix of the SCA3 group (left) and control group (right). **(C)** A SCA3 network (left) and a normal control network (right) constructed at the original *P*-value of 0.042.

In graph theoretical analysis, a brain network is defined by a collection of *n* nodes and *l* links, where the nodes represent the brain regions and the links represent structural, functional, or effective connections between pairs of brain regions (Friston, 1994). The linear regression was applied to remove the age and gender effects. The Pearson's correlation coefficients of 3D-FD of the paired cortical regions across participants were computed to produce an interregional correlation matrix for each group. Only positive correlation coefficients were retained to construct the brain networks (Figure 2A) and the negative correlation coefficients were set to zero, as suggested in a previous study (Rubinov and Sporns, 2010). Notably, each retained positive correlation coefficient had a *P*-value representing the significance of the correlation between paired regions.

In this study, we first set a *p*-value of 0.05 as a threshold to filter the connection matrix followed by selecting 20% of the largest correlation coefficients. After the process, the retained correlations were set to ones. In addition, all entries on the main diagonal (self-to-self connections) and negative coefficients were set to zeros. The binarized matrix was called a connection matrix, in which the value of 1 represented an existent link between a pair of regions and the value of 0 otherwise. Figure 2B demonstrates the final binary matrix (size: 97 × 97) in which 1 (white in Figure 2B) represents an existent link and 0 (black in Figure 2B) represents no link between a pair of regions. Figure 2C presents the visualized glass brain of network of connection matrix.

The size of the connection matrix was 97 × 97 in this study, which consisted of a maximum possible 4,656 (97 × 96/2) links between pairs of regions in the network. Let us define the set of all nodes as *N* and the number of nodes as *n*. A link between nodes *i* and *j* can be written as (*i, j*), where *i, j* ∈ *N* and the entry *a*_*ij*_ in the connection matrix represented the connection status (if the link between nodes *i* and *j* existed, *a*_*ij*_ = 1; *a*_*ij*_ = 0 otherwise). Subsequently, some network properties can be computed accordingly.

Graph topology such as largest component size, clustering coefficient, path length, and efficiency are often used to characterize network properties. In particular, degree and betweenness are employed to determine the critical areas within a network. Largest component size of a network is referred to the number of nodes in the largest connected component. Density of a network represents the fraction of links retained from the full network. Degree of a node *i* is defined as

(1) $$\begin{array}{r} k_{i}=\sum_{j\in N}a_{ij} \end{array}$$

Mean degree of the network is defined as

(2) $$\begin{array}{r} k=\frac{\sum_{i\in N}k_{i}}{n} \end{array}$$

Shortest path length between nodes *i* and *j* is defined as

(3) $$\begin{array}{r} d_{ij}=\sum_{a_{uv}\in g_{i↔j}}a_{uv} \end{array}$$

where *g*_*i*↔*j*_ is the shortest path (geodesic distance) between *i* and *j*. If the nodes *i* and *j* are not connected, *d*_*ij*_ = ∞. Number of triangles is defined as

(4) $$\begin{array}{r} t_{i}=\frac{1}{2}\sum_{j,h\in N}a_{ij}a_{ih}a_{jh} \end{array}$$

Betweenness centrality of node *i* (Freeman, 1978) is defined as

(5) $$b_{i}=\frac{1}{\left(n-1)(n-2\right)}\sum_{\begin{array}{l} \;h,j\;\in \;N\\ h\neq j,h\neq i,j\neq i \end{array}}\frac{\rho_{hj}(i)}{\rho_{hj}}$$

where ρ_*hj*_ is the number of shortest paths between *h* and *j*, and ρ_*hj*_(*i*) is the number of shortest paths between *h* and *j* that pass through *i*. In this study, the difference in nodal betweenness between SCA3 and normal control networks was investigated. In addition to largest component size, mean degree, density, we computed, and compared three important global network topological properties, namely, characteristic path length (*L*_*p*_) (Watts and Strogatz, 1998), clustering coefficient (*C*_*p*_) (Watts and Strogatz, 1998), and global efficiency (*E*_*glob*_) (Latora and Marchiori, 2001). Because the largest component size of the network was not always 97, which means that some distances between two nodes may be infinite, we used harmonic mean (Newman, 2003) to calculate *L*_*p*_ as follows

(6) $$L_{p}=\frac{1}{n}\sum_{i\in N}\frac{\sum_{j\in N,j\neq i}d_{ij}}{n-1}$$

which was a basis for measuring network

*C*_*p*_ is defined as

(7) $$\begin{array}{r} C_{p}=\frac{1}{n}\sum_{i\in N}\frac{2t_{i}}{k_{i}\left(k_{i-1}\right)} \end{array}$$

which is a measure of network segregation. *E*_*glob*_ is defined as

(8) $$E_{glob}=\frac{1}{n}{\sum^{\text{​}}}_{i\in N}\frac{\sum_{j\in N,j\neq i}d_{ij}}{\left(n-1\right)}$$

Besides, the characteristic path length, clustering coefficient, and global efficiency of the SCA3, and normal networks were also compared by the ones derived from random networks, which were created based on the rewiring procedure described by Maslov and Sneppen (2002). Network analysis was conducted by using brain connectivity toolbox (Rubinov and Sporns, 2010) on the MATLAB software.

### Analysis of Network Resilience: Random and Targeted Attacks

Network resilience represents the degree of tolerance of a network against random and targeted attacks (Albert et al., 2000; Achard et al., 2006; He et al., 2008). In this study, we measured the network resilience by removing nodes from the networks. For random attacks, we randomly removed one node after another from each network and calculated the changes in the characteristic path length, clustering coefficient, and global efficiency values, and largest component size. For targeted attacks, we repeated the aforementioned process but removed nodes in the descending order of their nodal betweenness. The difference in the network resilience against random and targeted attacks between the SCA3 and normal control networks was then investigated.

### Statistical Analysis

Note that we only obtained one 3D-FD value for each parcellated region. For each group with 40 subjects, there are 40 3D-FD values for each region. We computed the 3D-FD value based Pearson correlation between any two regions. As a result, a 97 by 97 correlation map was obtained for each group to build a structural network, resulting in one set of small-world properties for each structural network. Accordingly, we cannot directly perform any statistical comparison on the corresponding small-world properties between these two structural networks. To statistically compare the differences of network properties between the SCA3 and control groups, a permutation test was conducted (Bullmore et al., 1999). In the process, we set *P*-value range from 0.05 to 0.001 and the network properties at each *P*-value were computed for the SCA3 and control groups. To test the null hypothesis that network property differences between the groups occurred by chance, we randomly reassigned the SCA3 patients and healthy controls into two groups and recomputed the correlation matrix for each randomized group. This randomized simulation and recalculation of the network properties was repeated 1,000 times. The 95th percentile points of each distribution of the 1,000 simulations were used as critical values in a two-sample one-tailed *t*-test to reject the null hypothesis with a type I error probability of 0.05.

## Results

**Figure 3:**
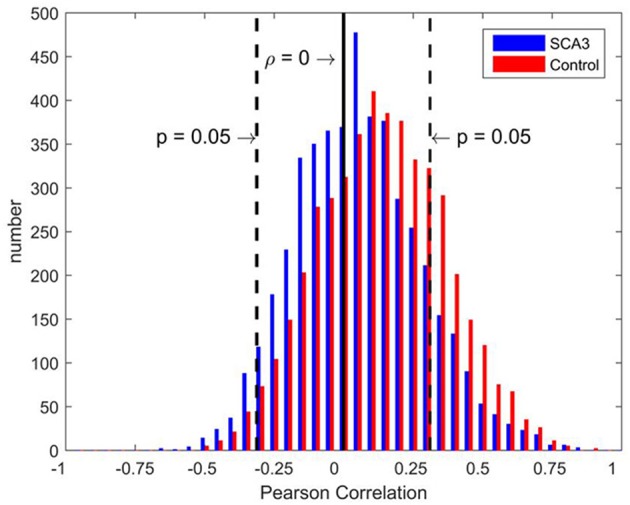
Bar chart of Pearson's correlation coefficients of the paired brain regions of SCA3 patients (blue) and normal controls (red). The black solid line represents a correlation coefficient of 0. Black dashed lines represent a significance level equivalent to *P* = 0.05.

Distributions of Pearson's correlation coefficients derived from the 3D-FD values of paired brain regions across the groups of SCA3 patients and normal controls are displayed in Figure 3. The distribution from normal controls (red bars in Figure 3) was close to a normal distribution, which had a kurtosis (Kim, 2013) of 3.263, skewness (Kim, 2013) of 0.992, and mean of 0.158. However, the distribution from SCA3 patients (blue bars in Figure 3) had a kurtosis of 5.439, skewness of 1.642 and mean of 0.105. The distribution from SCA3 patients demonstrates a left shift (smaller mean) compared to the one from normal controls. Moreover, normal controls had more significant positive correlations than SCA3 patients (red bars are much higher than corresponding blue bars in the right side beyond the dash line of *p*-value of 0.05 in Figure 3); whereas, SCA3 patients had more significant negative correlations in comparison with normal controls (blue bars are much higher than corresponding red bars in the left side beyond the dash line of *p*-value of 0.05 in Figure 3).

### Largest Component Size, Mean Degree, and Density of SCA3 and Normal Control Networks

**Figure 4:**
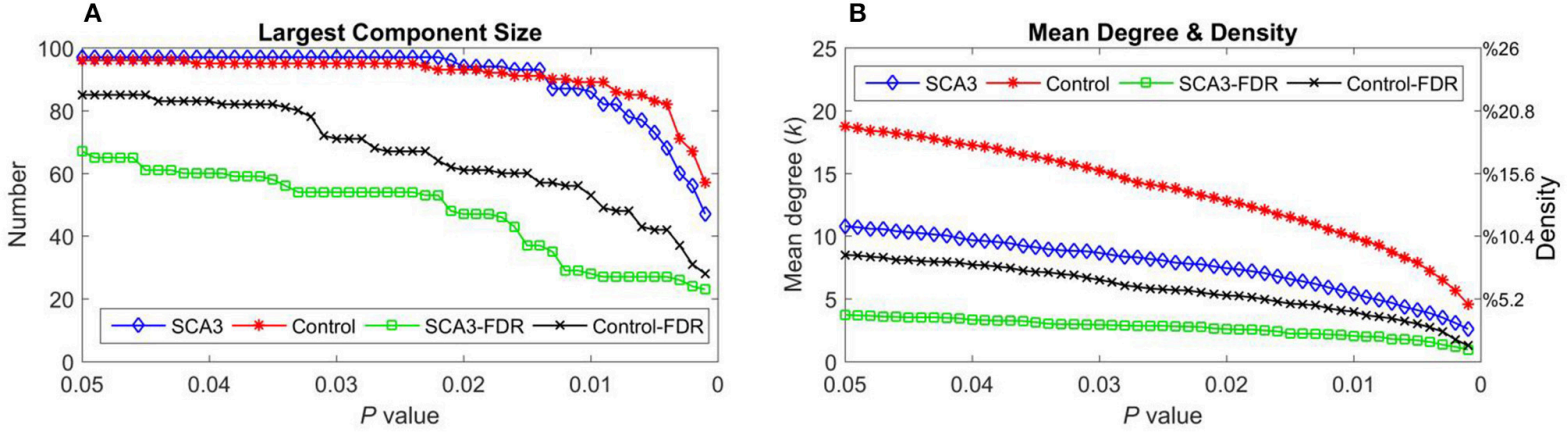
**(A)** Largest component size and **(B)** mean degree (left y-axis) and density (right y-axis) of the SCA3 and normal control networks at different *P*-values and FDR-corrected *P*-values. Blue diamonds (SCA3) indicate the parameters of the SCA3 networks at different *P*-values, and red stars (Control) indicate the parameters of the normal control networks at different *P*-values. Green squares (SCA3-FDR) indicate the parameters of the SCA3 networks at different FDR-corrected *P*-values. Black crosses (Control-FDR) indicate the parameters of the normal control networks at different FDR-corrected *P*-values.

**Figure 5:**
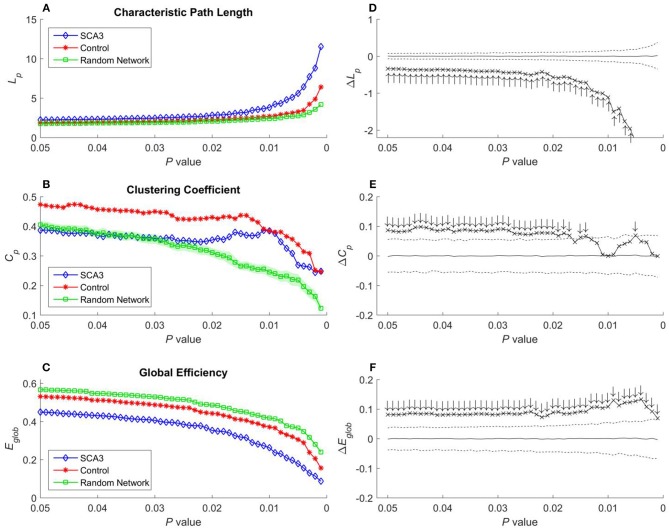
**(A)** Characteristic path length (*L*_*p*_), **(B)** clustering coefficient **(*C***_***p***_**)**, and **(C)** global efficiency (*E*_*glob*_) of the SCA3, normal control, and random networks at different *P*-values. Between-group difference (SCA3 vs. normal control) in the **(D)** characteristic path length values, **(E)** clustering coefficient values, and **(F)** global efficiency values. In the left column, blue diamonds (SCA3) indicate the properties of the SCA3 networks at different *P*-values, red stars (Control) indicate the properties of the normal control networks at different *P*-values, and green squares (Random Network) indicate the mean of the properties of simulated random networks at different *P*-values. The green shadows around the green lines indicate the standard deviation of the properties of the simulated random networks. In the right column, the crosses indicate the differences between the SCA3 and normal networks in the **(D)** characteristic path length (Δ*L*_*p*_) values, **(E)** clustering coefficient (Δ*C*_*p*_) values, and **(F)** global efficiency (Δ*E*_*glob*_) values at different *P*-values. Solid lines indicate the mean value of the 1,000 permutation results. Dashed lines indicate the 95% confidence intervals of the 1,000 permutation results, and ↑ and ↓indicate a significant difference.

Table 2 summarizes the network parameters, including the largest component size (LCS), mean degree (MDeg), mean density (MDen), characteristic path length (L_p_), clustering coefficient (C_p_), and global efficiency (E_glob_), between SCA3 and normal control groups at FDR-*p* value = 0.05. The SCA3 group presented decreased values in the LCS, mean degree, MDen, clustering coefficient, and global efficiency, and increased value in characteristic path length. These results revealed SCA3 group had a more sparsity and disrupted properties in their network. The graphs of the LCS, mean degree, density, characteristic path length, clustering coefficient, and global efficiency of the SCA3 and normal networks are displayed in Figures 4, 5. The LCSs of SCA3 and normal control networks of different *P-*values (0.05–0.001, permutation test) and FDR-corrected *P*-values (0.05–0.001, permutation test) are displayed in Figure 4A. The results of the mean degree and density of the SCA3 and normal control networks are displayed in the graph (Figure 4B), where the left and right y-axes represent the mean degree and density, respectively.

**Table 2:** Results of network properties between SCA3 and normal control groups at FDR-*p*-value = 0.05.

**Groups**	**LCS**	**MDeg**	**MDen%**	**L_**p**_**	**Cp**	**E_**glob**_**
NC	86	8.1	8.6%	4.3	0.36	0.32
SCA3	67	4.2	4.7%	7.2	0.27	0.17

*LCS, largest component size; MDeg, mean degree; MDen, mean density; L_p_, Characteristic Path length; C_p_, Clustering coefficient; E_glob_, Global efficiency*.

### Characteristic Path Length, Clustering Coefficient Values, and Global Efficiency Values

Characteristic path length, clustering coefficient, and global efficiency values of the SCA3 network, normal control network, and simulated random networks at *P*-values in the range 0.05–0.001 are displayed in Figures 5A–C, respectively.

In Figure 5A, the characteristic path length of the SCA3 networks (blue diamonds) show consistently higher values than those of normal control (red stars) and random networks (green squares). Furthermore, the normal control networks (red stars) consistently exhibit higher characteristic path length values than the random networks (green squares); however, their values are similar at each *P*-value. In Figure 5D, the differences (cross marks) in the characteristic path length values between the SCA3 and normal control networks are consistently located outside the 95th percentile (dashed line) in the simulated distribution created using the permutation test, thus indicating that the differences are significant at *P*-values in the range between 0.001 and 0.05.

In Figure 5B, the clustering coefficient values of the normal control networks (red stars) are considerably higher than those of the random networks (green squares) over the entire range of *P*-values. The clustering coefficient values of the SCA3 networks (blue diamonds) are very similar to those of the random networks (green squares), but they are smaller than the clustering coefficient values of the normal control networks at *P*-values in the range between 0.03 and 0.05. However, as the *p*-values become more significantly different (from 0.03 down to 0.001), the clustering coefficient values of the SCA3 networks (blue diamonds) remain relatively stable, starting to diverge from those of the random networks (green squares), and approaching those of the normal control networks (red stars). In Figure 5E, most differences in the clustering coefficient values between the SCA3 and normal networks are significant (most cross marks are outside the dashed curve representing the 95% confidence intervals).

**Figure 6:**
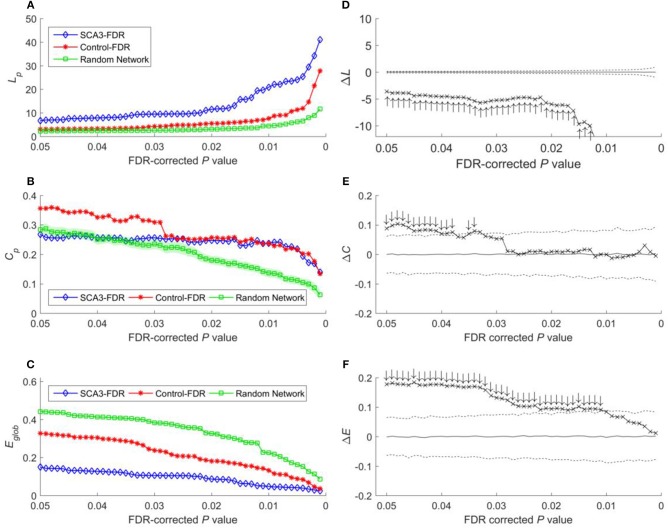
**(A)** Characteristic path length (*L*_*p*_), **(B)** clustering coefficient **(*C***_***p***_**)**, and **(C)** global efficiency (*E*_*glob*_) of the SCA3, normal control, and random networks at different FDR-corrected *P*-values. Between-group difference (SCA3 vs. normal control) in the **(D)** characteristic path length values, **(E)** clustering coefficient values, and **(F)** global efficiency values. In the left column, blue diamonds (SCA3) indicate the properties of the SCA3 networks at different FDR-corrected *P*-values, red stars (Control) indicate the properties of the normal control networks at different FDR-corrected *P*-values, and green squares (Random Network) indicate the mean of the properties of simulated random networks at different FDR-corrected *P*-values. The green shadows around the green lines indicate the standard deviation of the properties of the simulated random networks. In the right column, the crosses indicate the differences between the SCA3 and normal networks in the **(D)** characteristic path length (Δ*L*_*p*_) values, **(E)** clustering coefficient (Δ*C*_*p*_) values, and **(F)** global efficiency (Δ*E*_*glob*_) values at different FDR-corrected *P*-values. Solid lines indicate the mean value of the 1,000 permutation results. Dashed lines indicate the 95% confidence intervals of the 1,000 permutation results, and ↑ and ↓indicate a significant difference.

Because global efficiency correlated inversely with the characteristic path length, the results of the global efficiency analysis of the SCA3, normal control, and random networks exhibited an inverse relationship with those of characteristic path length. The normal control networks (Figure 5C, red stars) and random networks (Figure 5C, green squares) had higher global efficiency values than the SCA3 networks did (Figure 5C, blue diamonds), and the global efficiency values of the normal control and random networks were similar to each other. The results of the permutation test also revealed that the differences in the global efficiency values between the SCA3 and normal control networks were significant at *P*-values in the range between 0.001 and 0.05 (all cross marks are outside the dashed line in Figure 5F). Figure 6 illustrates the differences in the characteristic path length, clustering coefficient, and global efficiency values between the SCA3 and normal control networks remained significant at most thresholds of FDR-corrected *P*-values.

**Figure 7:**
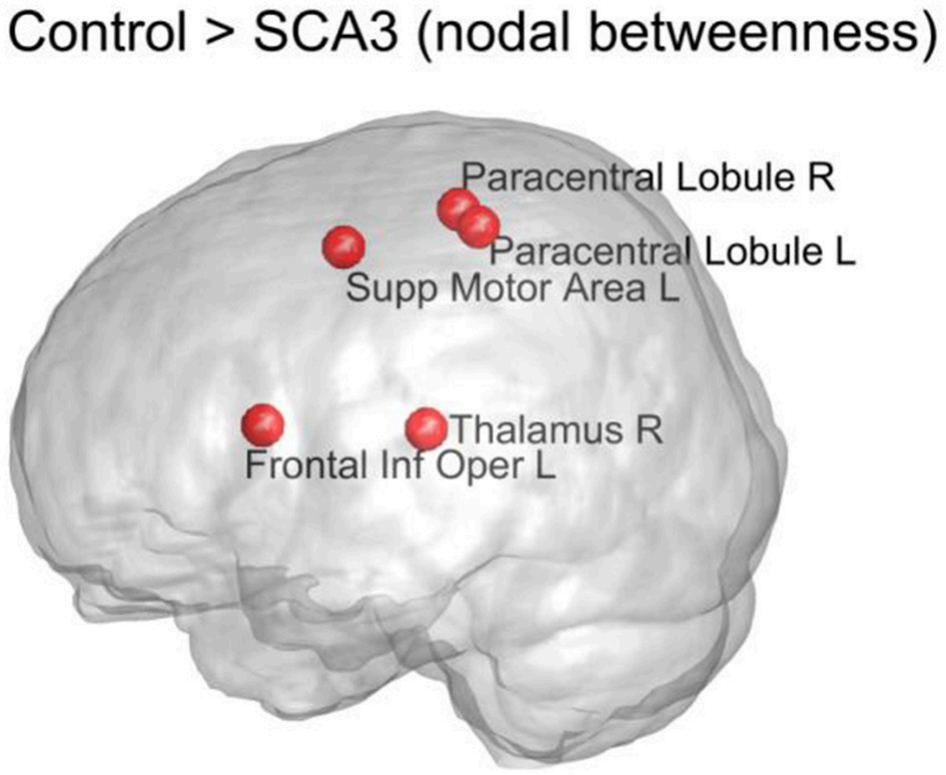
Top 5 nodes that had the largest significant decrease (*p* < 0.001) in nodal betweenness between the SCA3 and normal control networks. The statistical significance was obtained from 1,000 permutation tests. R, right; L, left; Supp, supplementary; Inf, inferior; Oper, operculum.

Figure 7 displays the top five nodes of largest reduced nodal betweenness in the SCA3 network compared to the corresponding nodes of the normal control network. The statistical significance of these five nodes (*p* < 0.001) was obtained from 1,000 permutation tests. The top five nodes in descending order were left supplementary motor area (Supp Motor Area L), right thalamus (Thalamus R), left frontal inferior operculum (Frontal Inf Oper L), right paracentral lobule (Paracentral Lobule R), and left paracentral lobule (Paracentral Lobule L).

### Resilience of SCA3 and Normal Control Networks

**Figure 8:**
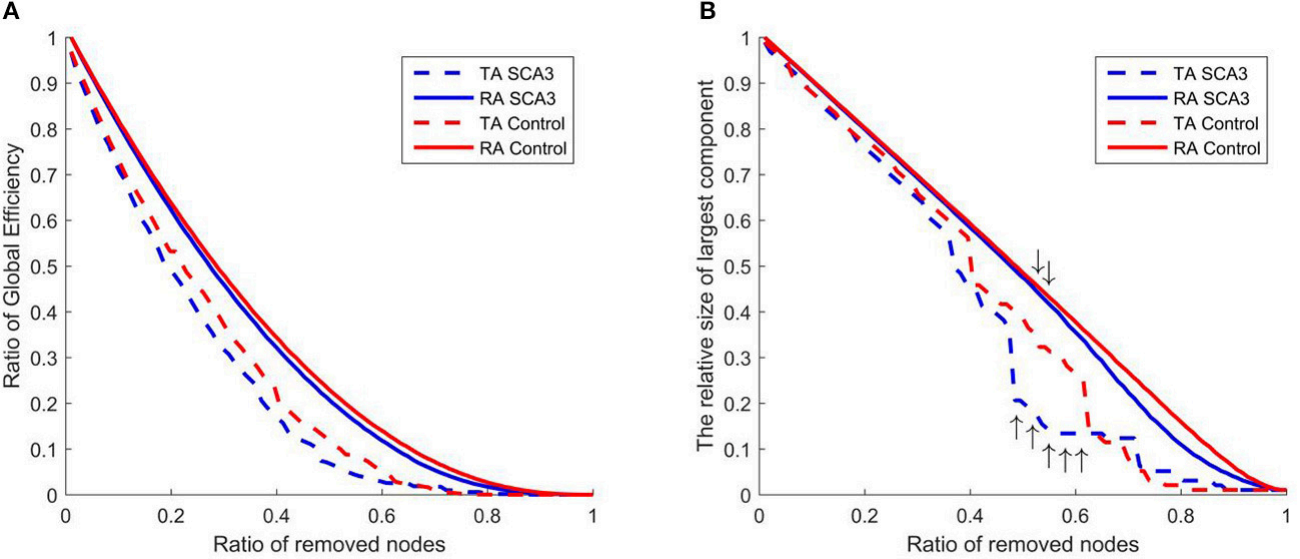
Evaluation of the resilience of the SCA3 networks (blue) and normal control networks (red) to random attacks (solid line) and targeted attacks (dashed line) based on **(A)** global efficiency value and **(B)** largest component size. The graphs indicate the ratio of global efficiency and the relative size of largest component as functions of the ratio of removed nodes in the random attack and targeted attack analyses. TA and RA indicate targeted attacks and random attacks, respectively.

Figure 8 displays the resilience of the normal control and SCA3 networks in response to random and targeted attacks. Although the SCA3 and normal control networks had similar resilience against random attacks, the SCA3 networks were more vulnerable to targeted attacks. The global efficiency values of the SCA3 networks were consistently lower than those of the normal control networks (Figure 8A) under random and targeted attacks, and most of the differences between the SCA3 and normal control networks in the global efficiency values were larger under targeted attacks than under random attacks (Figure 8A). In the relative size of the largest component, the SCA3 and normal control networks displayed similar responses to random attacks only until at least ~52% of nodes and their links were removed (Figure 8B, down arrows); subsequently, the SCA3 networks started exhibiting a greater reduction in the size of the largest component than the normal control networks did. Furthermore, when 48–61% of the most central nodes were attacked in the SCA3 and normal control networks (Figure 8B, up arrows), the relative sizes of largest component approached the largest difference, ~12–16%, between the two networks.

## Discussion

Since the introduction of the human connectome (Sporns et al., 2005), many studies have related neurodegenerative diseases to the human brain networks (Bassett and Bullmore, 2009). Alterations in structural networks have been reported in the patients with Alzheimer's disease (He et al., 2008; Kim et al., 2016), Parkinson's disease (Xu et al., 2017), and Huntington's disease (Coppen et al., 2016). He et al. (2007) used cortical thickness from MRI to build structural network of brain, and demonstrated the basic organizational principles for the anatomical network in the human brain compatible with previous functional networks studies, which provides important implications of how functional brain states originate from their structural underpinnings. They also showed the human brain anatomical network has robust small-world properties with cohesive neighborhoods and short mean distances between regions. The present study is the first to show that structural networks are affected by SCA3. Because the structural brain network correlates with the functional network (Fjell et al., 2017), the altered structural networks presented in this study might have underlying effects on the ataxia symptoms of SCA3 patients.

### Loss of Small-World Topology in SCA3 Networks

The brain structural network has been shown to have similar properties to those of a small-world network (He et al., 2007). The small-world, characterized by a high degree of clustering and short path length, is an attractive model for the description of complex brain networks because it not only supports both specialized and integrated information processing (Sporns and Zwi, 2004) but also minimizes wiring costs while maximizing the efficiency of information propagation (Kaiser and Hilgetag, 2006; Achard and Bullmore, 2007). The average of clustering coefficients over all nodes is the clustering coefficient of the network, often used as a global metric of the network level of segregation (Sporns, 2013). Small-world properties can only be estimated if the mean degree of a network is larger than the value of log (*N*) (*N* is the number of nodes in the network). In present study, most of the mean degrees of the SCA3 and normal control networks were higher than the value of log (97), that is 4.574, at *P*-values in the range between 0.001 and 0.05 (Figure 4B). This suggests that the constructed structural networks in the range of *P*-values should present a small-world architecture.

In this study, the characteristic path length values of the normal control networks were similar to those of the random networks; however, the normal control networks had considerably higher clustering coefficient values than did the random networks. This implies that the structural networks of the normal control networks based on cerebral 3D-FD values are likely to have small-world architecture. However, the SCA3 networks had considerably smaller characteristic path length values compared with the random networks. In addition, the clustering coefficient values of the SCA3 networks were highly similar to those of the random networks in the *P*-value range between 0.03 and 0.05, although the gaps between them became notable at *P*-values below 0.03 (Figure 5B). These results indicate that the SCA3 networks might have lost their small-world topology.

Loss of small-world topology in brain networks has also been associated with neurological and mental disorders including Alzheimer's disease (Stam et al., 2007; He et al., 2008), autism (Barttfeld et al., 2011), and schizophrenia (Liu et al., 2008; Yu et al., 2011; Wang et al., 2012b). The disorganization of small-world topology in SCA3 patients reflects the pathological state of the brain networks.

### Disrupted Structural Networks in SCA3

Apart from the loss of the small-world attribute, the SCA3 structural networks exhibited significantly higher values in the characteristic path lengths (Figures 5A,D), and significantly lower values in the clustering coefficients (Figures 5B,E), and global efficiency (Figures 5C,F) compared with the normal control networks. The SCA3 revealed a more sparsity and disrupted structural network with decreased values in the LCS, mean degree, MDen, clustering coefficient, and global efficiency and increased value in characteristic path length.

Deterioration of the topological parameters of the structural network has also been reported in Alzheimer's disease (He et al., 2008; Kim et al., 2016), Parkinson's disease (Xu et al., 2017), and Huntington's disease (Coppen et al., 2016). The reduced LCS in network of SCA3 may indicate the organization of structural networks shifts from a more distributed architecture in control group to a local anatomical emphasis in SCA3 patients. The cortico-cerebral circuits in SCA3 were disrupted and segregated into occipital-parietal (visual-spatial cognition) and frontal-pre-drontal (motor control) clusters. The cerebellum of SCA3 were segregated from cerebellum-temporal-frontal circuits and clustered into a frontal-temporal cluster (cognitive control). Therefore, the disrupted structural network presented in this study might reflect the clinical characteristics of SCA3.

Moreover, short paths in brain networks derived from cortical thickness assure effective integrity or rapid transfers of information between and across remotely regions that are believed to constitute the basis of cognitive processes (Sporns and Zwi, 2004). In Table 2, SCA3 showed increased path length, decreased degree of clustering, and global efficiency, suggesting their structural networks are from integration to segregation with loss of small-world properties.

The deteriorated network parameters in the SCA3 networks could be attributed to the left shift of the distribution of correlation coefficients, which reduced the values of several positive correlation coefficients to less than the *P*-value thresholds (Figure 3). It implies a general structural disruption between paired brain regions. The exact reason for the dissociation of structural networks in SCA3 patients is not known. However, several possible mechanisms, including diaschisis, transneuronal degeneration, and dedifferentiation, were reviewed in a recent study (Fornito et al., 2015).

The aforementioned left shift of the distribution not only reduced the number of significantly positive links but also slightly increased the number of significantly negative links in the SCA3 networks (Figure 3). The role of negative links in the functional brain networks was discussed in a recent study (Parente and Colosimo, 2016). However, to the best of our knowledge; research on the negative correlations of the structural attributes between paired brain regions has not been conducted. Neural compensation may partially explain the negative correlations (Fornito et al., 2015; Jones, 2017). However, further effort should be directed toward identifying the mechanisms underlying these correlations. In the present study, we only analyzed the positive links in the networks.

### Decrease of Nodal Betweenness in Motor Control-Related Regions in SCA3

The measure of betweenness centrality is related to communication processes, but is also often found to be highly correlated with the related measure of closeness, quantifying the proximity of each node to the rest of the network (Sporns, 2013). According to our results, the left supplementary motor area, bilateral paracentral lobules (encompassing parts of the motor and sensory cortices), and right thalamus of SCA3 patients exhibited a considerable reduction (*P* < 0.001) in nodal betweenness compared with the normal controls. Because the left supplementary motor area and bilateral paracentral lobules had significantly lower 3D-FD values, the atrophy in these regions is implied to be associated with the change in their roles in the structural network (Wang et al., 2015). We did not observe a significant decrease in the 3D-FD values of the right thalamus in our previous study (Wang et al., 2015). Therefore, the significant decrease in the betweenness of the right thalamus may have resulted from the altered neighboring network organization. In a previous study, the paracentral lobules in healthy individuals were classified as connector hubs (high degree and high betweenness) in the structural network derived from tractography on diffusion tensor images (Hagmann et al., 2008). Hence, the decline of nodal betweenness in bilateral paracentral lobules of SCA3 patients might have functional implications.

Rüb et al. (2008) first demonstrated that the neurodegeneration in localized brain regions of SCA3 patients could implicate dysfunction of several central nervous circuits and further lead to clinical symptoms. Tada et al. (2015) suggested that degenerative ataxias might be caused by damage to key nodes in the functional system of motor control, in which only the dentate nuclei, pontine nuclei, and Clarke's column were shown to be majorly involved in SCA3 (Farrar et al., 2016). However, a recent study based on transcranial magnetic stimulation reported that dysfunction of the motor cortex is an early feature of SCA3 and is associated with motor symptoms and ataxia (Seidel et al., 2012). Furthermore, the degeneration of the primary motor cortex (Rüb et al., 2008; Seidel et al., 2012; Wang et al., 2015), supplementary motor area (Wang et al., 2015), and thalamic ventral lateral nuclei (Seidel et al., 2012; Kang et al., 2014) are comprehensively reported in the literature. Reports from previous studies and the results of the present study suggest that the degeneration of pivotal regions in the corticopontocerebellar and dentatothalamic circuits might collectively contribute to the onset of SCA3 ataxia symptoms.

**Figure 9:**
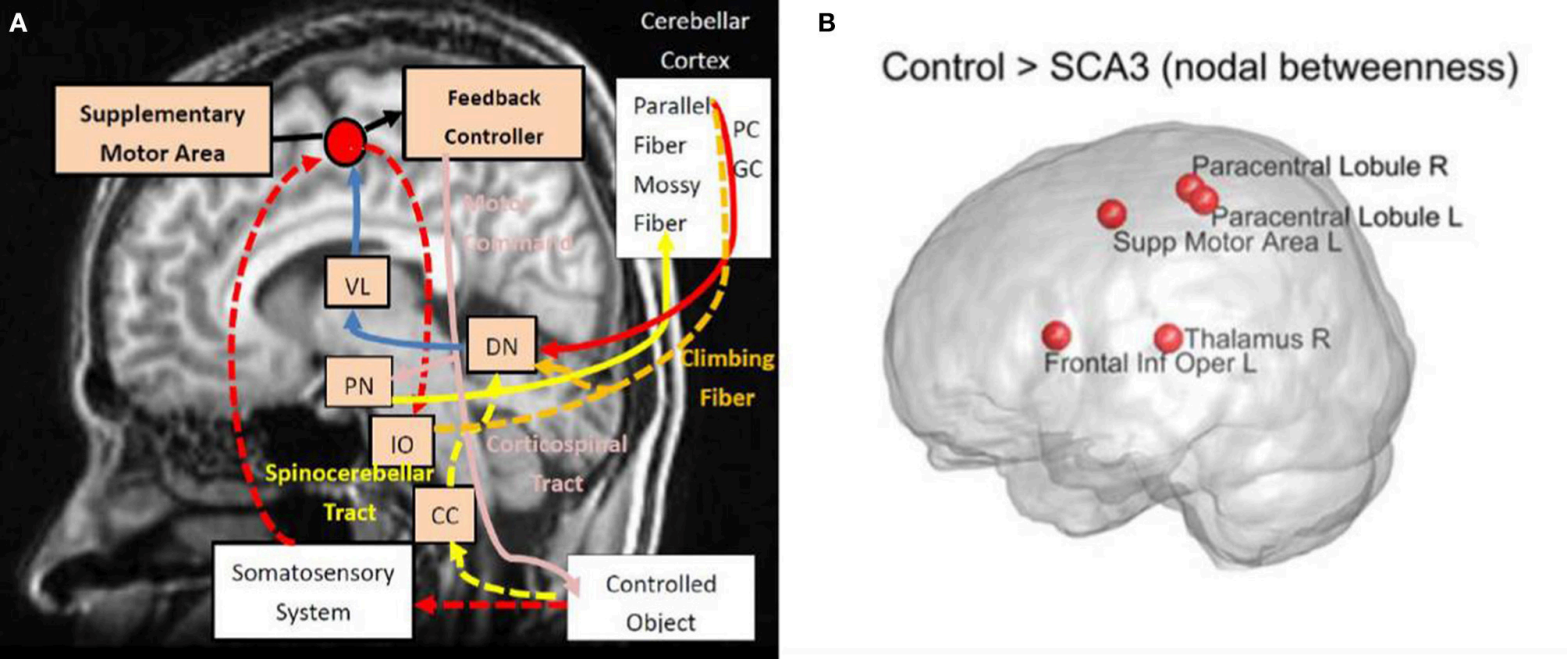
**(A)** Disease involvements in SCA3. **(B)** The nodes with reduced nodal betweenness in this study. Diagram **(A)** is modified from the one provided by Tada et al. (2015). VL, ventral lateral thalamus; PN, pontine nucleus; DN, dentate nucleus; IO, inferior olive; CC, Clarke's column; PC, Purkinje fibers; and GC, granule cell. Intensity of red color stands for the prominence of reported degeneration. R, right; L, left; Supp, supplementary; Inf, inferior; Oper, operculum.

A diagram (Figure 9A) is modified from a previous study by Tada et al. (2015) to illustrate in what regions of the motor control circuits the degeneration is involved. Compared Figure 9A with Figure 7 (re-displays in Figure 9B), we can easily observe that four of the top five nodes with the biggest reduction in nodal betweenness (left supplementary area, bilateral paracentral lobules, and right thalamus) in the SCA3 network are closely matched the disease-involved regions reported in the literature. It suggests that the degeneration of pivotal regions in the corticopontocerebellar and dentatothalamic circuits might collectively contribute to the onset of SCA3 ataxia symptoms.

### Impaired Network Resilience in SCA3 Networks

In this study, our results demonstrate that both the SCA3 and normal control networks are more vulnerable to targeted attacks than random attacks. This finding is in agreement with previous findings in human function based on functional network (Joyce et al., 2013) and in brain disorder on structural brain networks (He et al., 2008; Alstott et al., 2009; Crossley et al., 2014). Furthermore, the SCA3 networks were more vulnerable to targeted attacks than the normal control networks were. This finding implies that compared with the normal networks, the topology of SCA3 networks are disrupted and the disease inflicts more damage on the SCA3 networks if the central nodes are attacked. The reduced resilience of the SCA3 networks might be ascribed to the left shift in the distribution of correlation coefficients, loss of small-world architecture, and altered nodal betweenness centrality as mentioned previously. In summary, the SCA3 networks were found to be more vulnerable to targeted attacks than was the normal control network. The structural networks of the brains of SCA3 patients are disrupted by the disease. These findings suggest that future studies should consider the effects of global cerebral involvement in SCA3 patients.

However, the present study needs further investigation of correlation between the results of structural network and clinical data, such as clinical dementia rating scale (CDR) or functional MRI data. This may allow us to infer the changes of functional network from that of the structural network in patients with SCA3.

## Author Contributions

Y-TW organized the research project, reviewed and revised the manuscript. S-RH contributed the organization, statistical analysis, and wrote the first draft of the study. C-WJ performed the statistical analysis and revised the manuscript. B-WS organized the research and performed data collection. P-SW organized the research project, reviewed, and critiqued the manuscript. J-FL and H-MW accessed research data.

### Conflict of Interest Statement

The authors declare that the research was conducted in the absence of any commercial or financial relationships that could be construed as a potential conflict of interest.

## Footnotes

^1^Available online at: www.sph.sc.edu/comd/rorden/mricro.html

^2^Welcome Department of Cognitive Neurology. Institute of Neurology, University College London, London. Available online at: http://www.fil.ion.ucl.ac.uk/spm/

^3^Individual Brain Atlases using the Statistical Parametric Mapping. Available online at: http://www.thomaskoenig.ch/Lester/ibaspm.htm
